# Preservation Versus Routine Removal: Morbidity and Long‐Term Outcomes Following Diagnostic Laparoscopy Versus Negative Appendectomy—A Regional Multicentre Observational Cohort Study

**DOI:** 10.1002/wjs.70571

**Published:** 2026-09-19

**Authors:** Katrine Folmann Finne, Liv Hornnes, Anders Bang‐Nielsen, Aya Nihad Abdulrahman Abdulrahman, Talha Malik, Peter Cornelius Helbo Jakobsen, Lars Nannestad Jørgensen, Jakob Kleif, Claus Anders Bertelsen

**Affiliations:** ^1^ Department of Surgery Copenhagen University Hospital—North Zealand Hillerød Denmark; ^2^ Digestive Disease Center Copenhagen University Hospital—Bispebjerg Copenhagen Denmark; ^3^ Gastrounit Copenhagen University Hospital—Amager and Hvidovre Hvidovre Denmark; ^4^ Department of Surgery Copenhagen University Hospital—Herlev and Gentofte Herlev Denmark; ^5^ Department of Clinical Medicine Faculty of Health and Medical Sciences University of Copenhagen Copenhagen Denmark

## Abstract

**Background:**

Guidelines recommend laparoscopic appendectomy (LA) for a macroscopically normal appendix when no pathology is identified during diagnostic laparoscopy (DL) for suspected acute appendicitis (AA). This approach may lead to increased hospitalization, higher costs, and greater morbidity. Whether these guidelines are justified remains debatable.

**Methods:**

This retrospective cohort study included all patients aged > 2 years undergoing DL for suspected AA at four Danish University hospitals from March 2017 to April 2021. Patients undergoing LA despite normal intraoperative findings were compared with patients with normal intraoperative findings undergoing DL only. Sensitivity analyses included patients with incidental intraoperative gynecological or histopathological findings not corresponding with the symptoms. The primary outcome was subsequent DL for suspected AA during follow‐up, and the main secondary outcomes were complications within 30 days.

**Results:**

627 patients underwent DL and 518 underwent LA. Median follow‐up was 5.2 years (IQR 4.1–6.1) and 5.7 years (IQR 4.5–6.6), respectively. At a median of 1.7 years (IQR 0.7–3.4), 3.5% (95% CI 2.0–4.9) of the DL group underwent a second DL for suspected AA, with a subsequent 0.96% risk of AA. In the main analyses, no significant differences between the groups were observed for subsequent intra‐abdominal surgery, gastrointestinal endoscopy or surgical complications. In sensitivity analyses, surgical complications occurred significantly more frequently after LA (3.7% vs. 1.6% after DL, *p* = 0.02), mainly driven by a trend toward a higher risk of intra‐abdominal bleeding (0.6% vs. 0%, *p* = 0.056) and intra‐abdominal abscess.

**Conclusions:**

The risks of both subsequent DL and AA following initial DL are minimal. The risk of increased surgical complications after LA is low, and the clinical relevance is questionable.

## 1 Introduction

Acute appendicitis (AA) is the one of most common surgical emergencies worldwide. Whilst laparoscopic appendectomy (LA) is the gold standard treatment [1], the debate regarding resection of the appendix during a diagnostic laparoscopy (DL) with inconclusive findings persists. The European Association of Endoscopic Surgery (EAES) and the World Society of Emergency Surgery (WSES) recommend the routine removal of a macroscopically normal appendix if no other pathology is identified during a DL for suspected AA [1, 2].

In Denmark, the non‐inflamed appendix has traditionally been left in situ. Although no national recommendations exist, this approach has evolved, resulting in considerable variation in surgical strategy. The reliability of laparoscopic assessment is questioned: microscopic pathology is found in 27%–70% of macroscopically normal appendices [3, 4], yet is absent in 10%–36% of macroscopically inflamed cases [5, 6, 7, 8]. This diagnostic uncertainty contributes to significant variation in negative appendectomy rates [9]. Studies find DL alone associated with a very low risk of subsequent AA, leading to arguments against routine appendectomy in macroscopically normal cases [10, 11]. However, preservation of the appendix is linked to readmission and recurrent right iliac fossa pain, which constitutes a separate economic healthcare issue [10].

Given strong arguments supporting both surgical strategies, further research regarding postoperative morbidity is warranted. Using data from a regional cohort, this study aims to compare both short‐ and long‐term morbidity of preservation versus routine removal of the macroscopically normal appendix.

## 2 Methods

### 2.1 Study Design and Setting

This retrospective, multicentre cohort study was conducted at four public academic hospitals (Bispebjerg, Herlev, Hvidovre, and North Zealand), serving the 1.9 million inhabitants of the Capital Region of Denmark. All patients aged ≥ 2 years were included; children aged < 2 years were managed in a specialized pediatric department. Pediatric patients were not treated at Bispebjerg Hospital. In Denmark, all emergency abdominal surgery is performed in the public, tax‐financed healthcare system, with none performed in the private sector.

The inclusion period began on 17 March 2017 (Herlev, Hvidovre, North Zealand) and 20 May 2017 (Bispebjerg) and ended on 1 April 2021. Data were extracted from the electronic health record system by surgical residents, audited by the first and last authors, and included all public hospital contacts and pathology reports in the Capital Region of Denmark. To guarantee adequate surveillance for late complications, a minimum follow‐up threshold of 3 years from the index surgical procedure was established a priori. Given the rolling inclusion and sequential audit updates performed between April 1, 2024, and April 1, 2025, individual follow‐up durations and study closeout dates varied across the cohort. The study is reported in accordance with the STROBE guidelines [12].

Ethical approval and patient consent were not required under Danish law. Data collection was approved by the Danish Patient Safety Authority (#31‐1521‐283), the regional Data Protection Agency (P‐2020‐436), and respective departments, adhering to GDPR and Danish legislation. The study was registered on ClinicalTrials.gov (NCT06000020).

### 2.2 Diagnostics and Surgical Intervention

According to clinical practice in Denmark, the indication for diagnostic laparoscopy (DL) in suspected acute appendicitis (AA) is based on clinical presentation and laboratory findings (C‐reactive protein and white blood cell count) [13]. Preoperative imaging was performed at the treating surgeon's discretion, with ultrasonography preferred in pediatric patients when feasible. Contrast‐enhanced computed tomography (CT) was commonly performed in adults ≥ 50 years without recent colonoscopy, to rule out underlying colorectal malignancy. Radiological assessments were made by consulting radiologists according to local practice.

DL was standard first‐line management across all participating hospitals. In selected cases (e.g., repeated clinical presentations or pediatric patients), appendectomy could be performed despite the absence of macroscopic inflammation based on intraoperative assessment. In Denmark, DL for suspected AA is typically performed by surgical trainees under supervision, which was also the case in this study. All appendices resected underwent routine pathological assessment.

Prophylactic antibiotics are generally administered pre‐ or intraoperatively. Postoperative antibiotics, including administration for complicated appendicitis or signs of intra‐abdominal infection, are at the surgeon's discretion.

### 2.3 Participants and Cohort Definitions

Patients who underwent DL or LA for suspected AA were eligible for inclusion. The DL group (control group) comprised patients who a macroscopically normal appendix and no other intra‐abdominal pathology identified requiring further treatment. The LA group (study group) comprised patients who underwent appendectomy despite a macroscopically normal appendix. Histopathological examination confirmed absence of acute inflammation in the resected appendix. Mesenteric adenitis or non‐inflamed Meckel's diverticulum were considered non‐explanatory findings and did not require exclusion.

### 2.4 Variables

Preoperative variables included age, sex, hospital, body mass index, American Society of Anesthesiologists'(ASA) score, and previous surgical history. Intraoperative variables included operative findings as described by the surgeon, procedure type, length of surgery, antibiotic administration, and surgical injuries including their relation to laparoscopy or appendectomy. Postoperative variables included antibiotic treatment, length of stay, histopathology (if an appendectomy was performed), readmission within 30 days, and medical and surgical complications including reintervention within 30 days. AA was histologically defined as acute inflammation with the transmural presence of polymorphonuclear leukocytes [14].

### 2.5 Outcomes

The primary outcome was the cumulative incidence of a subsequent DL or laparotomy for suspected AA, with a minimum follow‐up of 3 years from index surgery. Main secondary outcome was the proportion of patients in the DL group with histologically proven AA after the index surgery. Other secondary outcomes included postoperative complications (according to the Clavien‐Dindo classification), length of stay, readmission, and mortality within 30 days from index surgery. Long‐term outcomes included subsequent intra‐abdominal or endoscopic gastrointestinal procedures.

### 2.6 Sensitivity Analyses

Post‐hoc sensitivity analyses of the outcomes were performed. Patients with histopathological fibrous obliteration of the appendix or parasites in a normal appendix were included. Furthermore, in both groups, female patients with incidental gynecological findings were also included. These findings were defined as: simple ovarian cysts smaller than 4 cm; paraovarian and paratubal cysts; corpus luteum cysts (without rupture or bleeding); fibromas; fluid collection in the rectouterine pouch; and suspected salpingitis, provided that no antibiotic was subsequently administered.

### 2.7 Statistical Analysis

Continuous data are presented as median and interquartile ranges (IQR), and categorical data, as frequencies and proportions. The Kruskal‐Wallis test and Fisher's exact test were used as appropriate. The rate ratios of DL and LA between hospitals were analyzed by Poisson regression and presented with 95% confidence intervals (CI). All available data were used, and no imputation was performed for missing data. Analyses were performed using R statistical software version 4.5.0 (R Foundation for Statistical Computing, Vienna, Austria) [15].

### 2.8 Role of the Funding Source

The study was not funded. KFF and CAB had access to all data, and as corresponding author, KFF had the final responsibility for submission.

## 3 Results

During the study period, 10,512 patients underwent DL for suspected AA at the four participating hospitals: Bispebjerg (*n* = 1947); Herlev (*n* = 3126); Hvidovre (*n* = 3204); and North Zealand (*n* = 2235). The median age was 31 years (IQR 21–47; range 2–99), and 5689 (54.1%) patients were female.

Patient inclusion is shown in Figure 1. The main analyses included 627 patients in the DL group and 518 patients in the LA group. Tables 1, 2, 3 to 3 summarize group allocation, baseline characteristics, and postoperative outcomes, respectively. One patient in the DL group died from a small bowel injury unrecognized during the index surgery; this complication was not related to the decision not to perform an appendectomy.

**FIGURE 1:**
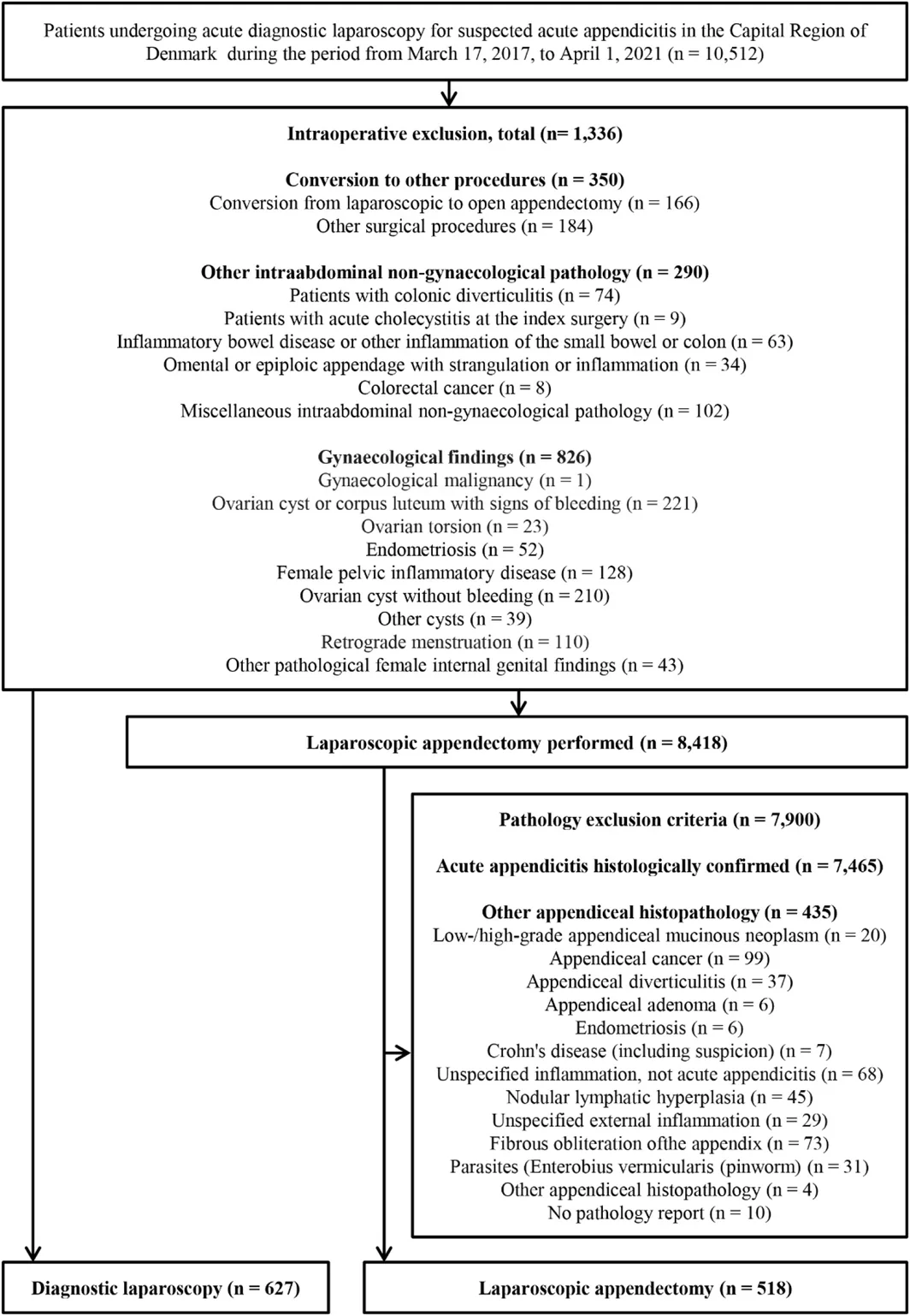
Flow‐chart of inclusion and exclusion of patients.

**TABLE 1:** Proportions of patients undergoing diagnostic laparoscopy and negative laparoscopic appendectomy, respectively, stratified by hospital. Poisson regression models with the hospital with the largest case volume (Hvidovre) as reference.

Hospital	Proportion of screened patients (%)	Rate ratio (95% c.i.)	*p* value
Patients included in the diagnostic laparoscopy group			
Hvidovre	176 of 3204 (5.5%)	1	
Bispebjerg^a^	76 of 1947 (3.9%)	0.71 (0.54–0.93)	0.013
Herlev	243 of 3126 (7.8%)	2.40 (2.04–2.83)	< 0.001
North Zealand	132 of 2235 (5.9%)	1.08 (0.86–1.35)	0.529
Patients included in the laparoscopic appendectomy group			
Hvidovre	27 of 3204 (0.8%)	1	
Bispebjerg^a^	44 of 1947 (2.3%)	2.68 (1.66–4.33)	< 0.001
Herlev	233 of 3126 (7.5%)	8.85 (5.94–13.2)	< 0.001
North Zealand	214 of 2235 (9.6%)	11.36 (7.61–17.0)	< 0.001

*Note:* Data are presented as *n of N* (%), rate ratio (95% c.i), Poisson regression models.

^a^ Bispebjerg does not provide pediatric surgical service and gynecology service.

**TABLE 2:** Demographics, comorbidity, surgical history, and intraoperative variables, stratified by diagnostic laparoscopy or laparoscopic appendectomy.

Variables	Diagnostic laparoscopy	Laparoscopic appendectomy
	(*N* = 627)	(*N* = 518)
Age, median [IQR]—years	22 [15–29]	20 [14–28]
Sex—no. no. (%) [95% CI]		
Female	416 (66%) [62%–70%]	299 (58%) [53%–62%]
Male	211 (34%) [30%–38%]	219 (42%) [38%–47%]
BMI, median [i.q.r.]—kg/m^2,^ ^a^	23 [20–26]	22 [18–25]
ASA physical status—no. (%) [95% CI]		
1	434 (69%) [65%–73%]	375 (72%) [68%–76%]
2	177 (28%) [25%–32%]	137 (26%) [23%–31%]
3–4	16 (2.6%) [1.5%–4.2%]	6 (1.2%) [0.47%–2.6%]
Previous abdominal surgery—no. (%) [95% CI]	57 (9.1%) [7.0%–12%]	59 (11%) [8.8%–15%]
Preoperative imaging performed—no. (%) [95% CI]	133 (21%) [18%–25%]	56 (11%) [8.3%–14%]
Preoperative CT scan performed—no. (%) [95% CI]	34 (5.4%) [3.8%–7.6%]	21 (4.1%) [2.6%–2%]
CT scans showing acute appendicitis	15 of 34 (44%) [28%–62%]	11 of 21 (52%) [30%–74%]
Preoperative ultrasonography performed—no. (%) [95% CI]	105 (17%) [14%–20%]	36 (6.9%) [5.0%–9.6%]
USG showing acute appendicitis—no. (%) [95% CI]	13 of 105 (12%) [7.0%–21%]	13 of (36%) [21%–54%]
Intraoperative findings		
Macroscopically normal appendix—no. (%) [95% CI]	300 (48%) [44%–52%]	63 (12%) [9.5%–15%]
Acute appendicitis—no. (%) [95% CI]	0	412 (80%) [76%–83%]
Mesenteric adenitis with macroscopically normal appendix—no. (%) [95% CI]	343 (55%) [51%–59%]	115 (22%) [19%–26%]
Operation time, median [IQR]—minutes	31 [25–39]	45 [34–59]
Perioperative administration of antibiotics—no. (%) [95% CI]	136 (22%) [19%–25%]	481 (93%) [90%–95%]

*Note:* Data are presented as *n of N* (%)*, median (i.q.r.)*.

^a^ Missing data on body mass index (BMI) in 185 patients.

**TABLE 3:** Postoperative complications within 30 days, length of stay, and readmission rate, stratified by diagnostic laparoscopy or laparoscopic appendectomy.

Variables	Diagnostic laparoscopy	Laparoscopic appendectomy
	(*N* = 627)	(*N* = 518)
Injury to other organ (recognized during surgery)—no. (%)	1 (0.2%)	2 (0.4%)
Small bowel—no. (%)	1 (0.2%)	0
Colon—no. (%)	0	1 (0.2%)
Other organ—no. (%)	0	1 (0.2%)
Surgical complications—no. (%)	12 (1.9%)	18 (3.5%)
Intra‐abdominal abscess—no. (%)	0	2 (0.4%)
Wound infection—no. (%)	4 (0.6%)	2 (0.4%)
Wound infection (Clavien‐Dindo 3b–)—no. %	1	0
Intra‐abdominal bleeding (all Clavien‐Dindo 3b–)—no. %	0	2 (0.4%)
Small bowel perforation diagnosed after index surgery—no. (%)	1 (0.2%)	0
Port hernia (all Clavien‐Dindo 3b–)—no. (%)	1 (0.2%)	1 (0.2%)
Wound bleeding—no. %	6 (1.0%)	10 (1.9%)
Wound bleeding (Clavien‐Dindo 3b–)—no. %	0	2 (0.4%)
Severe surgical complication (Clavien–Dindo 3b–5)—no. (%)	4 (0.6%)	5 (1.0%)
Non–surgical complications—no. (%)	4 (0.6%)	6 (1.2%)
Pneumonia—no. (%)	0	2 (0.4%)
Urinary tract infection—no. (%)	2 (0.3%)	0
Urinary retention—no. (%)	0	1 (0.2%)
Other non–surgical complications—no. (%)	2 (0.3%)	3 (0.6%)
Severe non–surgical complication (Clavien–Dindo 3b–5)—no. (%)	1 (0.2%)	0
Length of stay, median [i.q.r.]—hours	10 [5–17]	9 [4–15]
Readmission—no. (%) [95% CI]	84 (13%) [11%–16%]	85 (16%) [13%–20%]
Time before readmission, median [i.q.r.]—days	2.8 [0.6–8.4]	3.4 [0.7–8.5]

*Note:* Data are presented as *n* (%), *median (i.q.r.)*. Missing data on body mass index (BMI) in 185 patients.

The median follow‐up was 5.2 years (IQR 4.1–6.1) for the DL group and 5.7 years (IQR 4.5–6.6) for the LA group. Within 30 days postoperatively, one patient in each group died from causes related to preoperative comorbidities. Additionally, two patients were lost to follow‐up due to emigration shortly after the index operation. Complete follow‐up data were available for the remaining 1140 patients.

After a median time of 1.7 years (IQR 0.7–3.4), 22 patients (3.5%; 95% CI 2.0–4.9) in the DL group underwent subsequent DL for suspected AA. Of these, 21 patients underwent LA, with six having histologically proven AA. The risk of subsequent AA in the DL group was 0.96%. Table 4 shows the demographics, intraoperative findings, and histopathology. During follow‐up, the incidence rate of AA in the DL group was 193 per 100,000 person‐years (95% CI 71–419).

**TABLE 4:** Patient demographics, intraoperative findings, and histopathology in the diagnostic laparoscopy group that underwent subsequent diagnostic laparoscopy for suspected acute appendicitis.

Variables	Subsequent diagnostic laparoscopy
	(*N* = 22)
Median age [range; i.q.r.]—years	17 [8–57; 12–19]
Adolescents (< 18 years old)—no. (%)	12 (55)
Sex (female:male ratio)—no. (%)	18 (82):4 (18)
Intraoperative findings	
Macroscopically normal appendix—no. (%)	7 (32)
Appendicitis—no. (%)	5 (28)
Mesenteric adenitis—no. (%)	5 (28)
Retrograde menstruation—no. (%)	2 (9)
Ovarian cyst without bleeding or rupture—no. (%)	3 (14)
Ovarian cyst with bleeding or rupture—no. (%)	1 (5)
Endometriosis—no. (%)	1 (5)
Histopathology of the appendix if appendectomy was performed—no. (%)	
Acute appendicitis—no. (%)	5 of 21 (24)
Microscopically normal appendix—no. (%)	14 of 21 (67)
Nodular lymphoid hyperplasia—no. (%)	1 of 21 (5)
Unspecified inflammation, not acute appendicitis—no. (%)	1 of 21 (5)

*Note:* Data are presented as *n* (%)*, median (i.q.r.; range)*.

One case with gangrenous appendicitis occurred less than 48 hours post‐index procedure; the apparently normal appendix had been preserved. The rate of severe complications (Clavien‐Dindo grade 3b or higher) related to the subsequent surgical procedure was 9%, mainly involving wound infection and port site bleeding. Table 4 demonstrates demographics, intraoperative findings, and histopathology of the 22 patients who underwent subsequent DL for suspected AA. Table 5 reports the types of follow‐up surgery and endoscopic procedures stratified by the index procedure.

**TABLE 5:** Subsequent abdominal surgeries and endoscopic gastrointestinal procedures for diagnoses other than suspected acute appendicitis, stratified by diagnostic laparoscopy or laparoscopic appendectomy at index procedure.

Variables	Diagnostic laparoscopy	Laparoscopic appendectomy	*p* value
	(*N* = 627)	(*N* = 518)	
Diagnostic laparoscopy—no. (%)	5 (0.8)	2 (0.4)	0.467
Incidental appendectomy during a gynecological procedure– no. (%)	0	0	NA
Colon resection—no. (%)	0	3 (0.6)	0.092
Small bowel resection—no. (%)	0	1 (0.2)	0.452
Caesarean section—no. (%)	25 (4.0)	21 (4.1)	1.000
Ovarian resection or oophorectomy—no. (%)	1 (0.2)	5 (1.0)	0.097
Salpingectomy—no. (%)	6 (1.0)	6 (1.2)	0.777
Hysterectomy or other transabdominal uterine procedure—no. (%)	4 (0.6)	3 (0.6)	1.000
Colonoscopy (≥ 1)– no. (%)	41 (6.5)	38 (7.3)	0.640
Gastroscopy (≥ 1)– no. (%)	20 (3.2)	30 (5.8)	0.041
Incisional hernia repair after index procedure—no. (%)	1 (0.2)	3 (0.6)	0.334
Other hernia repair—no. (%)	3 (0.5)	5 (1.0)	0.479
Adhesiolysis due to obstruction—no. (%)	1 (0.2)	0	1.000
Cholecystectomy—no. (%)	5 (0.8)	5 (1.0)	0.762
Operation for late complication (e.g. suture granuloma or surgical site infection) after postoperative day 30—no. (%)	3 (0.5)	2 (0.4)	1.000
Other abdominal or gastrointestinal endoscopic procedure—no. (%)	10 (1.6)	6 (1.2)	0.618

*Note:* Data are presented as *n* (%), Fisher's exact test.

### 3.1 Sensitivity Analyses

The sensitivity analyses included 736 patients in the DL group and 699 in the LA group. Supporting Information S1: Tables 1–2 summarize baseline characteristics, and postoperative outcomes, respectively. The respective cumulative incidence of postoperative intra‐abdominal bleeding requiring reoperation in the DL and LA groups were 0 (0%) and 4 (0.6%), *p* = 0.056.

Twenty‐nine patients in the DL group underwent subsequent DL for suspected AA. Sensitivity analyses identified one patient with histopathologically proven AA at the subsequent procedure, whereas six female patients in the DL group underwent LA without histological evidence of AA. The risk of subsequent AA in the DL group was 0.8%. Supporting Information S1: Table 3 shows the demographics, intraoperative findings, and histopathology findings of these 29 patients. Rates of subsequent abdominal surgery or endoscopic gastrointestinal procedures were comparable to the primary results (Supporting Information S1: Table 4).

## 4 Discussion

This study demonstrated a 3.5% risk of a subsequent laparoscopy or laparotomy for suspected AA in patients undergoing DL. Histopathologically confirmed AA was diagnosed in only 0.96% of patients undergoing subsequent DL after appendix‐preserving DL. The primary analysis did not demonstrate a difference in surgical complications between groups. In the sensitivity analysis including patients with minor incidental findings not considered explanatory for symptoms a higher rate of surgical complications was observed in the LA group; however, this broader, more heterogeneous study population should be interpreted with caution. The primary analysis remains the main basis for interpretation of the comparative outcomes.

The literature regarding risk of AA after DL is sparse and includes a limited number of patients [10, 11, 16]. However, our findings are consistent with those of another Danish cohort study including 271 patients whose appendix was left in situ, which reported a subsequent DL rate of 8.1%, while only 0.4% of patients had histopathologically confirmed AA [10]. Interestingly, they reported a readmission rate of 20.7%, representing significant healthcare costs. In comparison, 13.1% of patients in our DL group were readmitted. Additionally, this previous cohort was 81.6% female, whereas 66.3% of patients in the DL group and 62.2% of the LA group were female in this study.

Rates of routine LA varied between participating departments, with a lower rate of non‐inflamed appendices in patients admitted to two of four hospitals (Bispebjerg and Hvidovre). Confounding factors may influence this, as Bispebjerg does not provide pediatric surgery or gynecological services. Bispebjerg and Hvidovre have traditionally preferred an observational approach with repeated clinical assessments prior to DL. Previous studies [6, 11, 17] indicate a higher risk of negative appendectomy in females and younger patients, with rates reaching 39%–43% in the in women of childbearing age [6, 18]. This is likely due to the range of gynecological disorders mimicking AA in females presenting with right iliac fossa pain. One study reported that more than half of removed macroscopically normal appendices in women who underwent LA had abnormal histopathology other than acute inflammation, and advocated for increased use of DL to avoid overlooking symptomatic abdominal pathology [19].

Our lower risk of subsequent DL compared with previous findings may be partly due to increased use of preoperative imaging [10]. International studies show an inverse association between preoperative imaging and negative appendectomy [20, 21, 22, 23]. Specifically, CT‐scans in female patients are highly predictive with regard to appendiceal histopathology, suggesting this diagnostic approach included in the preoperative workup to reduce rates of unnecessary DL and LA in this specific population [18].

The four surgical departments participating did not routinely use imaging for patients with classic clinical presentation of AA. WSES guidelines recommend cross‐sectional imaging before surgery in adult patients with persisting or progressive clinical symptoms, noting that contrast‐enhanced low‐dose CT scan can aid in diagnosing young adults and adolescents, although laparoscopy is still recommended [24]. While one recent study [25] found selective CT use reduced DL to 4.2% in adults, it included an initial abdominal ultrasound: a strategy difficult to apply uniformly within the Danish setting. Furthermore, evidence is conflicting. An Australian study [26] supports the selective preoperative imaging strategy, as they found no significant difference in histopathological appendicitis rates between mandatory or selective CT, while a recent study surprisingly found no association between CT use and negative appendectomy rate [27].

Current guidelines by EAES [1] and WSES [24] maintain the recommendation to remove a normal‐appearing appendix if no other intra‐abdominal disease is found, due to the potentially unreliable macroscopic assessment by the surgeon. However, the recommendation is widely based on retrospective studies with small sample sizes. One study [16], based on 259 laparoscopically removed appendices, found that AA was microscopically verified in 12 out of 42 macroscopically normal specimens. The same study reported that none presented with similar symptoms or required surgery at 6‐month follow‐up among those 18 patients (6%) whose appendix was left in situ. Strong et al. [3] conducted a prospective multicentre study including almost 2000 laparoscopic appendectomies, and found that in 375 macroscopically normal appendices, 26.6% had microscopic pathological findings. Pathological examination predominantly revealed acute inflammation, with approximately 25% of specimens showing non‐inflammatory pathology. Conversely, when the surgeon assessed the appendix as inflamed, histopathological analysis was normal in 9.6% of cases.

The guidelines stress the risk of overlooking microscopic inflammation intraoperatively. However, the definition of inflammation may vary and therefore influence the rate of negative appendectomy or pathologic histopathological findings [28]. In agreement with other studies [19, 29, 30], non‐specific inflammation, nodular lymphoid hyperplasia, parasite infiltration, and lumen obliteration were revealed in the present study (Figure 1). It remains unclear if these histopathological findings correspond to the clinical symptoms. A recent study [31] examined a cohort of almost 3500 pediatric patients with clinical AA and reported a 7.4% rate of histopathological finding of lymphoid hyperplasia with no inflammatory cell infiltration. The study concluded that a more conservative management of these patients may be considered.

Previous studies have emphasized the postoperative morbidity associated with negative appendectomy [32]. Studies find postoperative complication rates following negative LA similar to LA for uncomplicated appendicitis, some as high as 6%–7% [4, 5, 6, 7, 8, 9, 10, 11]. In contrast, our results demonstrated lower postoperative complications rates in patients undergoing DL and LA. Sensitivity analysis revealed that surgical complications occurred more than twice as frequent in the LA group than in the DL group, however, despite statistical significance, the small absolute risk difference limits the clinical relevance of this finding.

## 5 Strengths and Limitations

This study has several limitations. The decision to leave an apparently normal appendix in situ or perform LA was at the discretion of the operating or supervising surgeon, rendering surgical practice subject to bias. Furthermore, some surgical centers traditionally offered LA regardless of intraoperative findings when a patient was booked for a second diagnostic laparoscopy for suspected AA.

The intraoperative macroscopic assessment of the appendix was a potential confounder. Lack of experience among junior surgeons may lead to misinterpretation of a secondarily injected, hyperemic or stiff appendix when found with concurrent intra‐abdominal pathology such as retrograde menstruation or salpingitis. In this study, gynecological pathology was excluded from the main analyses. Sensitivity analyses including patients with minor incidental gynecological findings did not materially change the overall interpretation of the primary findings, although differences between analyses should be considered when interpreting the results.

Data quality depended on descriptive skills and concise record keeping. All data were collected for the study, rather than retrieved from registers. To counter misclassification bias, the data set underwent multiple data audits by the first and second author, and any unusual cases were discussed within the group. Selection bias was minimized as all emergency surgery is performed within the public healthcare system in Denmark, giving investigators full access to medical records, resulting in a comprehensive data set and almost complete follow‐up.

A similar prospective study would be preferable, but we consider the feasibility questionable, as the same surgeon factor mentioned would remain present, though a standardized way to make the surgical report might reduce misclassification bias. Despite these limitations, the study design allowed for a large cohort of more than 10,500 patients with extensive follow‐up.

The incident rate of AA in Denmark is 275 (205–365) per 100,000 person‐years [33] compared with 193 (95% CI 71–419) per 100,000 person‐years in this study.

In this regional cohort, preservation of the macroscopically normal appendix was not associated with increased short‐ or long‐term morbidity compared with routine removal. The potential benefit of eliminating subsequent AA should be weighed against the risk of postoperative complications when a macroscopically normal appendix is observed during DL. Preoperative diagnostic imaging should be considered in patients with recurrent lower right‐sided abdominal pain after a previous negative DL.

## Author Contributions

**Katrine Folmann Finne:** investigation, writing – original draft, validation, writing – review and editing, project administration, data curation. **Liv Hornnes:** writing – review and editing, investigation, data curation, validation. **Anders Bang‐Nielsen:** conceptualization, investigation, writing – review and editing. **Aya Nihad Abdulrahman Abdulrahman:** writing – review and editing, investigation. **Talha Malik:** writing – review and editing, investigation. **Peter Cornelius Helbo Jakobsen:** writing – review and editing, investigation. **Lars Nannestad Jørgensen:** writing – review and editing, supervision. **Jakob Kleif:** conceptualization, writing – review and editing, formal analysis, methodology, data curation. **Claus Anders Bertelsen:** conceptualization, writing – review and editing, project administration, formal analysis, methodology, investigation, data curation, validation, resources.

## Funding

The authors have nothing to report.

## Ethics Statement

Approval from the Ethics Committee and patient consent is not needed under Danish legislation. The Danish Patient Safety Authority (#31‐1521‐283), the regional Data Protection Agency (P‐2020‐436), and the respective departments have approved data collection. Data management was conducted in accordance with the General Data Protection Regulation (GDPR) and Danish legislation. The study was registered on ClinicalTrials.gov (NCT06000020).

## Conflicts of Interest

The authors declare no conflicts of interest.

## Supporting information


Supporting Information S1

## Data Availability

After de‐identification, individual participant data will be made available to investigators who provide a methodologically sound proposal for meta‐analyses. Proposals should be directed to cabertelsen@gmail.com.
