# Supplementary material for: Preservation Versus Routine Removal: Morbidity and Long‐Term Outcomes Following Diagnostic Laparoscopy Versus Negative Appendectomy—A Regional Multicentre Observational Cohort Study
Source: World J Surg. 2026 Sep 19;50(10):2917–26. doi: 10.1002/wjs.70571 (PMC13662507; doi:10.1002/wjs.70571)
Supplement: Supplementary file 1 [file WJS-50-2917-s001.docx]

### **Title**

Preservation versus Routine Removal: Morbidity and long-term outcomes following diagnostic laparoscopy versus negative appendectomy: a regional multicentre observational cohort study

### **Authors**

Katrine Folmann Finne, MD^1^; (ORCID: 0009-0001-3192-3022)

Liv Hornnes, MD^1^; (ORCID: 0009-0005-0797-1989)

Anders Bang-Nielsen, MD^1^; (ORCID: 0000-0002-4393-866X)

Aya Nihad Abdulrahman Abdulrahman, MD^3,4^; (ORCID: 0009-0003-7900-7036)

Talha Malik, MD^2^; (ORCID: 0000-0002-9169-1622)

Peter Cornelius Helbo Jakobsen, MD^1^; (ORCID: 0000-0003-4796-9030)

Lars Nannestad Jørgensen, DMSc MD^3,5^; (ORCID: 0000-0001-7465-5374)

Jakob Kleif, PhD MD^1,4,5^; (ORCID: 0000-0002-9330-0410)

Claus Anders Bertelsen, PhD MD^1,5^; (ORCID: 0000-0001-6337-8447)

### **Affiliations**

1 Department of Surgery, Copenhagen University Hospital – North Zealand, Dyrehavevej 29, 3400, Hillerød, Denmark

2 Department of Surgery, Copenhagen University Hospital – Herlev and Gentofte, Borgmester Ib Juuls Vej 1, 2730, Herlev, Denmark

3 Digestive Disease Center, Copenhagen University Hospital – Bispebjerg, Bispebjerg Bakke 23, 2400, Copenhagen, Denmark

4 Gastrounit, Copenhagen University Hospital – Amager and Hvidovre, Kettegård Alle 36, 2650, Hvidovre, Denmark

5 Department of Clinical Medicine, Faculty of Health and Medical Sciences, University of Copenhagen, Blegdamsvej 3, 2200, Copenhagen, Denmark

### **Corresponding author**

Dr. Katrine Folmann Finne, Department of Surgery, Copenhagen University Hospital – North Zealand, Dyrehavevej 29, 3400, Hillerød, Denmark. Email: [Katrine.folmann.finne@regionh.dk](mailto:Katrine.folmann.finne@regionh.dk)

**Supplementary Materials - Index**

| **Supplementary Figures and Tables** |  |
| --- | --- |
| Supplementary Table 1 | *Page 3* |
| Supplementary Table 2 | *Page 5* |
| Supplementary Table 3  Supplementary Table 4 | *Page 7*  *Page 8* |

## Supplementary Table 1

Demographics, comorbidities, surgical history, and intraoperative variables of the patients included in the sensitivity analyses, stratified by diagnostic laparoscopy or laparoscopic appendectomy.

| Variables | Diagnostic laparoscopy | Laparoscopic appendectomy |
| --- | --- | --- |
|  | **(N = 736)** | **(N = 699)** |
| Median age [IQR] – years* | 22 [17–30] | 21 [14–30] |
| Sex – no. no. (%) [95% CI] |  |  |
| Female | 525 (71%) [68–75%] | 435 (62%) [59–66%] |
| Male | 211 (29%) [25–32%] | 264 (38%) [34–41%] |
| Median BMI [IQR] – kg/m^2^* | 23 [20–26] | 22 [19–26] |
| ASA physical status –(% [95% CI] |  |  |
| 1 | 503 (68%) [65–72%] | 494 (71%) [67–74%] |
| 2 | 215 (29%) [26–33%] | 191 (27%) [24–31%] |
| 3–4 | 18 (2.4%) [1.5–3.9%] | 14 (2.0%) [1.1–3.4%] |
| Previous abdominal surgery – no. (%) [95% CI] | 72 (10%) [8–12%] | 100 (14%) [12–17%] |
| Preoperative imaging performed – no. (%) [95% CI] | 155 (21%) [18–24%] | 92 (13%) [11–16%] |
| Preoperative CT scan performed – no. (%) [95% CI] | 39 (5.3%) [3.8–7.2%] | 42 (6.0%) [4.4–8.1%] |
| CT scans showing acute appendicitis | 15 of 39 (38%) [24–55%] | 24 of 42 (57%) [41–72%] |
| Preoperative ultrasonography performed– no. (%) [95% CI] | 123 (17%) [14–20%] | 53 (7.6%) [5.8–9.9%] |
| USG showing acute appendicitis – no. (%) [95% CI] | 16 of 123 (13%) [8–21%] | 16 of 53 (30%) [19–45%] |
| Intraoperative findings |  |  |
| Macroscopically normal appendix – no. (%) [95% CI] | 375 (51%) [47–55%] | 97 (14%) [11–17%] |
| Appendicitis – no. (%) [95% CI]) | 0 | 528 (76%) [72–79%] |
| Mesenteric adenitis with macroscopically normal appendix – no. (%) [95% CI] | 379 (51%) [48–55%] | 145 (21%) [18–24%] |
| Ovarian cyst (without bleeding or rupture) – no. (%) [95% CI] | 379 (51%) [48–55%] | 145 (21%) [18–24%] |
| Endometriosis – no. (%) [95% CI] | 2 (0.3%) [0.05–1.1%] | 4 (0.6%) [0.18–1.6%] |
| Suspected salpingitis (no subsequent treatment) – no. (%) [95% CI] | 4 (0.5%) [0.17–1.5%] | 2 (0.3%) [0.05–1.1%] |
| Paraovarian cyst – no. (% [95% CI] | 22 (3.0%) [1.9–4.6%] | 14 (2.0%) [1.1–3.4%] |
| Other incidental gynaecological finding – no. (%) [95% CI] | 22 (3.0%) [1.9–4.6%] | 14 (2.0%) [1.1–3.4%] |
| Median operation time [IQR] – minutes* | 31 [25–40] | 46 [35–60] |
| Preoperative or intraoperative antibiotics – no. (%) [95% CI] | 160 (22%) [19–25%] | 645 (92%) [90–94%] |

Data are presented as *n of N (%), median (i.q.r.)*. Missing data on BMI in 230 patients.

## Supplementary Table 2

Postoperative complications within 30 days, length of stay, and readmission rate for the sensitivity analyses cohort, stratified by diagnostic laparoscopy or laparoscopic appendectomy. Also includes pathological diagnoses of removed appendices following a negative appendectomy.

| Variables | Diagnostic laparoscopy | Laparoscopic appendectomy |
| --- | --- | --- |
|  | **(N = 736)** | **(N = 699)** |
| Injury to other organ (recognised during surgery) – no. (%) [95% CI] | 1 (0.1) | 5 (0.7) |
| Small bowel – no. (%) [95% CI] | 1 (0.1) | 0 |
| Caecum – no. (%) [95% CI] | 0 | 2 (0.3%) |
| Other part of the colon – no. (%) [95% CI] | 0 | 1 (0.1%)  1 (0.1%) |
| Internal female reproductive organs – no. (%) [95% CI] | 0 | 1 (0.1%) |
| Other organ – no. (%) [95% CI] | 0 | 1 (0.1%) |
| Surgical complications – no. (%) |  |  |
| Intra-abdominal abscess – no. (%) | 0 | 3 (0.4%) |
| Wound infection – no. (%) | 4 (0.5%) | 3 (0.4%) |
| Wound infection (Clavien-Dindo 3b–) – no. % | 1 | 1 |
| Intra-abdominal bleeding (all Clavien-Dindo 3b–) – no. % | 0 | 4(0.6%) |
| Small bowel perforation diagnosed after index surgery – no. (%) | 1 (0.1%) | 0 |
| Port hernia (all Clavien-Dindo 3b–) – no. (%) | 1 (0.1%) | 2 (0.3%) |
| Postoperative wound bleeding – no. % | 6 (0.8%) | 12 (1.7%) |
| Wound bleeding (Clavien-Dindo 3b–) – no. % | 0 | 2 (16.7%) |
| Severe surgical complication (Clavien–Dindo 3b–5) – no. (%) | 4 (0.5%) | 9 (1.3%) |
| Non–surgical complications – no. (%) | 8 (1.1%) | 9 (1.3%) |
| Pneumonia – no. (%) | 0 | 3 (0.4%) |
| Urinary tract infection – no. (%) | 4 (0.5%) | 1 (0.1%) |
| Urinary retention – no. (%) | 1 (0.1%) | 1 (0.1%) |
| Other non–surgical complications – no. (%) | 3 (0.4%) | 5 (0.7%) |
| Severe non–surgical complication (Clavien–Dindo 3b–5) – no. (%) | 1 (0.1%) | 0 |
| Median length of stay [IQR] – hours | 10 [4–17] | 9 [4–15] |
| Readmission – no. (%) [95% CI] | 96 (13%) [11–15%] | 115 (16%) [14–19%] |
| Median days to readmission [IQR] – days | 2.7 [0.56–8.3] | 2.9 [0.6–8.5] |
| Pathology of the appendix (only the appendectomy group) |  |  |
| Normal appendix – no. (%) [95% CI] | – | 616 (88%) [86–91%] |
| Fibrous obliteration of the appendix – no. (%) [95% CI] | – | 81 (12%) [9–14%] |
| Parasites in normal appendix – no. (%) [95% CI] | – | 2 (0.3%) [0– 0.42%] |
| Parasites in normal appendix – no. (%) [95% CI] | – | 2 (0.3%) [0– 0.42%] |

Data are presented as *n (%), median (i.q.r.)*.

## Supplementary Table 3

Demographics, intraoperative findings, and histopathology of the 29 patients in the diagnostic laparoscopy group from the sensitivity analyses who underwent subsequent diagnostic laparoscopy for suspected acute appendicitis.

| Variables | Subsequent diagnostic laparoscopy |
| --- | --- |
|  | **(N = 29)** |
| Median to subsequent diagnostic laparoscopy for suspected appendicitis [IQR] – years | 1.5 [0.7–3.6] |
| Median age [IQR; range] – years | 17 [13–19; 8–21] |
| Adolescents (< 18 years old) – no. (%) | 12 (55%) |
| Sex (female:male ratio) – no. (%) | 25 (86%):4 (14%) |
| Intraoperative findings |  |
| Macroscopically normal appendix – no. (%) | 10 (34%) |
| Appendicitis – no. (%) | 7 (24%) |
| Mesenteric adenitis – no. (%) | 6 (21%) |
| Retrograde menstruation – no. (%) | 3 (10%) |
| Ovarian cyst without bleeding or rupture – no. (%) | 6 (21%) |
| Ovarian cyst with bleeding or rupture – no. (%) | 1 (3%) |
| Endometriosis – no. (%) | 1 (3%) |
| Suspected salpingitis, no subsequent treatment – no. (%) | 1 (3%) |
| Paraovarian cyst – no. (%) | 1 (3%) |
| Histopathology of the appendix if appendectomy was performed – no. (%) |  |
| Acute appendicitis – no. (%) | 6 of 28 (21%) |
| Microscopically normal appendix – no. (%) | 19 of 28 (68%) |
| Nodular lymphoid hyperplasia – no. (%) | 2 of 28 (7%) |
| Unspecified inflammation, not acute appendicitis – no. (%) | 1 of 28 (4%) |

Data are presented as *n (%), median (i.q.r.; range)*.

## Supplementary Table 4

| Variables | Diagnostic laparoscopy | Laparoscopic appendectomy | P value† |
| --- | --- | --- | --- |
|  | **(N = 736)** | **(N = 699)** |  |
| Diagnostic laparoscopy – no. (%) | 5 (0.7%) | 5 (0.7%) | 1.000 |
| Incidental appendectomy during a gynaecological procedure – no. (%) | 1 (0.1%) | 0 | 1.000 |
| Colon resection – no. (%) | 0 | 3 (0.4%) | 0.115 |
| Small bowel resection – no. (%) | 0 | 1 (0.1%) | 0.487 |
| Caesarean section – no. (%) | 28 (3.8%) | 24 (3.4%) | 0.778 |
| Ovarian resection or oophorectomy – no. (%) | 2 (0.3%) | 5 (0.7%) | 0.276 |
| Salpingectomy – no. (%) | 8 (1.1%) | 6 (0.9%) | 0.790 |
| Hysterectomy or other transabdominal uterine procedure – no. (%) | 7 (1.0%) | 3 (0.4%) | 0.344 |
| Colonoscopy (≥1) – no. (%) | 46 (6.2%) | 52 (7.4%) | 0.403 |
| Gastroscopy (≥1) – no. (%) | 24 (3.3%) | 40 (5.7%) | 0.029 |
| Incisional hernia repair after index procedure – no. (%) | 2 (0.3%) | 5 (0.7%) | 0.276 |
| Other hernia repair – no. (%) | 3 (0.4%) | 5 (0.7%) | 0.496 |
| Adhesiolysis due to obstruction – no. (%) | 1 (0.1%) | 0 | 1.000 |
| Cholecystectomy – no. (%) | 9 (1.2%) | 5 (0.7%) | 0.424 |
| Operation for late complication (e.g. suture granuloma or surgical site infection) after postoperative day 30 – no. (%) | 3 (0.4%) | 4 (0.6%) | 0.719 |
| Other abdominal or gastrointestinal endoscopic procedure – no. (%) | 12 (1.6%) | 9 (1.3%) | 0.664 |

Sensitivity analyses of postoperative abdominal surgeries and endoscopic gastrointestinal procedures for diagnoses other than suspected acute appendicitis, stratified by diagnostic laparoscopy or laparoscopic appendectomy at index procedure.

Data are presented as *n (%),* Fisher's exact test.
